# Glutamate dehydrogenase activator BCH stimulating reductive amination prevents high fat/high fructose diet-induced steatohepatitis and hyperglycemia in C57BL/6J mice

**DOI:** 10.1038/srep37468

**Published:** 2016-11-22

**Authors:** Seung Jin Han, Sung-E Choi, Sang-A Yi, Jong Gab Jung, Ik-Rak Jung, Maureen Shin, Seok Kang, Hyunhee Oh, Hae Jin Kim, Dae Jung Kim, Ji Eun Kwon, Cheol Soo Choi, Kwan Woo Lee, Yup Kang

**Affiliations:** 1 Department of Endocrinology and Metabolism, Ajou University School of Medicine, Suwon, Gyunggi-do, 443-749, Republic of Korea; 2 Department of Physiology, Ajou University School of Medicine, Suwon, Gyunggi-do, 443-749, Republic of Korea; 3 Department of Biomedical Science, The Graduate School Ajou University, Ajou University School of Medicine, Suwon, Gyunggi-do, 443-749, Republic of Korea; 4 National Efficacy Evaluation Center for Metabolic Disease Therapeutics, Lee Gil Ya Cancer and Diabetes Institute, Inchon, 406-840, Republic of Korea; 5 Department of Pathology, Ajou University School of Medicine, Suwon, Gyunggi-do, 443-749, Republic of Korea; 6 Department of Internal Medicine, Gachon University Gil Medical Center, Incheon, 405-760, Republic of Korea

## Abstract

Individuals with non-alcoholic fatty liver disease (NAFLD) and type 2 diabetes (T2D) induced by high calorie western diet are characterized by enhanced lipogenesis and gluconeogenesis in the liver. Stimulation of reductive amination may shift tricarboxylic acid cycle metabolism for lipogenesis and gluconeogenesis toward glutamate synthesis with increase of NAD+/NADH ratio and thus, ameliorate high calorie diet-induced fatty liver and hyperglycemia. Stimulation of reductive amination through glutamate dehydrogenase (GDH) activator 2-aminobicyclo-(2,2,1)-heptane-2-carboxylic acid (BCH) reduced both *de novo* lipogenesis and gluconeogenesis but increased the activities of sirtuins and AMP-activated kinase in primary hepatocytes. Long-term BCH treatment improved most metabolic alterations induced by high fat/high fructose (HF/HFr) diet in C57BL/6J mice. BCH prevented HF/HFr-induced fat accumulation and activation of stress/inflammation signals such as phospho-JNK, phospho-PERK, phospho-p38, and phospho-NFκB in liver tissues. Furthermore, BCH treatment reduced the expression levels of inflammatory cytokines such as TNF-α and IL-1β in HF/HFr-fed mouse liver. BCH also reduced liver collagen and plasma levels of alanine transaminase and aspartate transaminase. On the other hand, BCH significantly improved fasting hyperglycemia and glucose tolerance in HF/HFr-fed mice. In conclusion, stimulation of reductive amination through GDH activation can be used as a strategy to prevent high calorie western diet-induced NAFLD and T2D.

Non-alcoholic fatty liver disease (NAFLD) is a manifestation of metabolic syndrome and strongly associated with insulin resistance and type 2 diabetes (T2D). NAFLD encompasses a range of conditions from simple hepatic triacylglycerol (TG) accumulation (steatosis) to severe inflammatory steatosis (steatohepatitis) that result in cirrhosis^1^. Studies in rodents and humans have revealed that hepatic TG accumulation is mainly caused by an overflow of fatty acids from lipolysis of TG in adipocytes and excessive *de novo* lipogenesis (DNL)^2^. Activation of lipogenic pathway and subsequent accumulation of TG increases cellular concentrations of various deleterious lipid intermediates that can eventually trigger hepatic insulin resistance^3^,^4^,^5^. In a state of insulin resistance induced by high fat/high carbohydrate western diet, high level of insulin and glucose up-regulates the transcription of lipogenic genes through sterol regulatory element binding protein-1c (SREBP-1c) and carbohydrate-responsive element-binding protein (CHREBP), respectively^6^. In addition, insulin resistance inhibits insulin-induced suppression of gluconeogenic genes such as phosphoenolpyruvate carboxykinase and glucose-6 phosphatase, and ultimately induces fasting hyperglycemia^7^.

In livers of individuals with NAFLD and T2D, anaplerotic reaction for refilling tricarboxylic acid (TCA) cycle intermediates should be activated since the TCA cycle intermediates such as citrate and oxaloacetate are used as substrates of DNL and gluconeogenesis, respectively^8^,^9^ (Supplementary Fig. 1). However, excessive stimulation of both DNL and gluconeogenesis induces relative deficiency of the TCA cycle intermediates, therefore accelerating ketogenic metabolism. On the other hand, individual metabolic pathway of DNL and gluconeogenesis is supposed to counteract each pathway because activation of each cataplerotic pathway consumes TCA cycle intermediate pool. In fact, it has been reported that inhibition of gluconeogenesis can lead to liver steatosis whereas repression of lipid synthesis can promote gluconeogenesis^10^,^11^. Recently, it has also been reported that loss of ketogenesis stimulated acetyl-CoA disposal through the TCA cycle, resulting in increased DNL and gluconeogenesis^12^.

Glutamate dehydrogenase (GDH) catalyzes the “reversible” dehydrogenation of glutamate to alpha-ketoglutarate and ammonia with NAD+ as a cofactor. High levels of GDH have been found in the mammalian liver, kidney, brain, and pancreatic islets. GDH plays a role in energy homeostasis and ammonia detoxification through oxidative deamination and reductive amination, respectively^13^ (Supplementary Fig. 1). The activity of this enzyme is regulated positively by allosteric activators, such as ADP and leucine and negatively by inhibitors, such as GTP, ATP, and palmitoyl-CoA^14^ (Supplementary Fig. 1). When the energy potential is low or amino acids are abundant in a meal, the oxidative deamination reaction is preferred for production of energy or disposal of ammonia^13^. GDH activity is thought to be suppressed by GTP and SIRT4-induced ADP-ribosylation when the energy potential through oxidation metabolism of nutrients is sufficient^15^,^16^. In particular, it has been reported that oxidative deamination plays an important role in amino acid-induced insulin secretion in beta cells^17^. On the other hand, oxidative deamination and reductive amination in hepatic GDH is bidirectional^18^,^19^,^20^. 2-Aminobicyclo-(2,2,1)-heptane-2-carboxylic acid (BCH) is a non-metabolized analogue of leucine that acts as a strong allosteric activator of GDH^17^.

While oxidative deamination replenishes TCA cycle intermediates, reductive amination may siphon TCA cycle intermediates into amino acid synthesis (Supplementary Fig. 1). Therefore, stimulation of reductive amination may reduce DNL and gluconeogenesis possibly due to insufficient supply of TCA cycle intermediates into DNL and gluconeogenesis. On the other hand, NADH is oxidized to NAD+ during reductive amination reaction. Since an increased level of NAD+/NADH ratio is required for the activation of various sirtuins (SIRTs) and AMP-activated kinase (AMPK)^21^,^22^ and decreased NAD+/NADH ratio is associated with a high incidence of high fat-induced metabolic disorders and the aging process^23^,^24^, stimulation of reductive amination might be able to reduce high calorie western diet-induced fatty liver and insulin resistance through activation of the SIRT/AMPK/PGC-1α axis.

The objective of this study was to determine whether GDH activator BCH can stimulate the reductive amination reaction under conditions of high energy potential, thus reducing DNL and gluconeogenesis but activating SIRTs and AMPK in hepatocytes. This study also determined whether BCH treatment could improve high fat/high fructose (HF/HFr) western diet-induced metabolic alterations such as hepatic steatosis, steatohepatitis, and hyperglycemia in male C57BL/6J mice.

## Results

### GDH activator BCH stimulated reductive amination in primary hepatocytes

**Figure 1:**
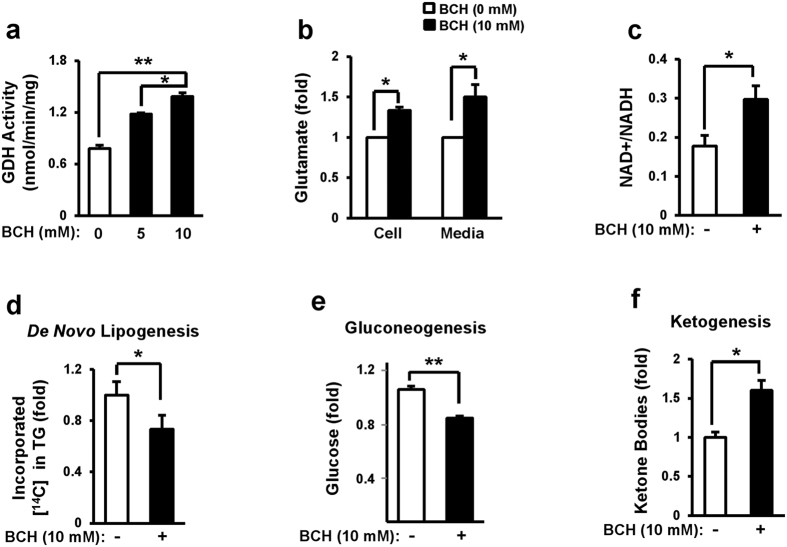
Stimulation of reductive amination through treatment with glutamate dehydrogenase activator BCH reduced *de novo* lipogenesis and gluconeogenesis while enhanced ketogenesis in primary hepatocytes. (**a**) GDH activity, (**b**) glutamate level, and (**c**) NAD+/NADH ratio were measured in primary hepatocytes treated with BCH for 12 h using respective assay kits. (**d**) *De novo* lipogenesis was determined by measuring incorporation of D-[U-^14^C]-glucose into triacyglycerol (TG). (**e**) Glucose production from lactate and pyruvate was determined by measuring the amount of glucose released from hepatocytes. (**f**) Ketogenesis was determined by measuring the amount of the released ketone bodies. Data were represented as means ± SEM of at least three independent experiments. ^*^p < 0.05, ^**^p < 0.01 vs. BCH-untreated group.

To determine whether activation of GDH could stimulate cataplerotic reductive amination in hepatocytes, primary hepatocytes isolated from normal C57BL/6J were incubated with GDH activator BCH for 12 h and the levels of glutamate and NAD+/NADH ratio were determined. BCH treatment significantly increased GDH activity in a dose-dependent manner (Fig. 1a). Cellular and extracellular levels of glutamate increased 38% and 50%, respectively, by 10 mM BCH (Fig. 1b). Cellular NAD+/NADH ratio was also elevated by 1.7-fold in BCH-treated hepatocytes compared to that in untreated control (Fig. 1c). These data suggest that BCH treatment stimulates reductive amination reaction in primary hepatocytes. On the other hand, BCH stimulated oxidative deamination in beta cells since BCH treatment reduced levels of glutamate and NAD+/NADH ratio in INS-1 cells (Supplementary Fig. 2a and b). Since stimulation of reductive amination might reduce DNL and gluconeogenesis, glucose incorporation into fat and glucose production from lactate and pyruvate were investigated in BCH-treated primary hepatocytes. BCH treatment resulted in significant reduction of both glucose incorporation into hepatic lipid (Fig. 1d) and glucose production from lactate and pyruvate (Fig. 1e). Since excessive stimulation of reductive amination can induce relative deficiency of TCA cycle intermediates, BCH treatment may induce ketogenesis. As shown in Fig. 1f, the levels of extracellular ketone bodies were significantly increased by the BCH treatment, supporting that GDH activator BCH stimulated reductive amination in cultured hepatocytes.

### BCH increased activities of SIRTs and AMPK in primary hepatocytes

**Figure 2:**
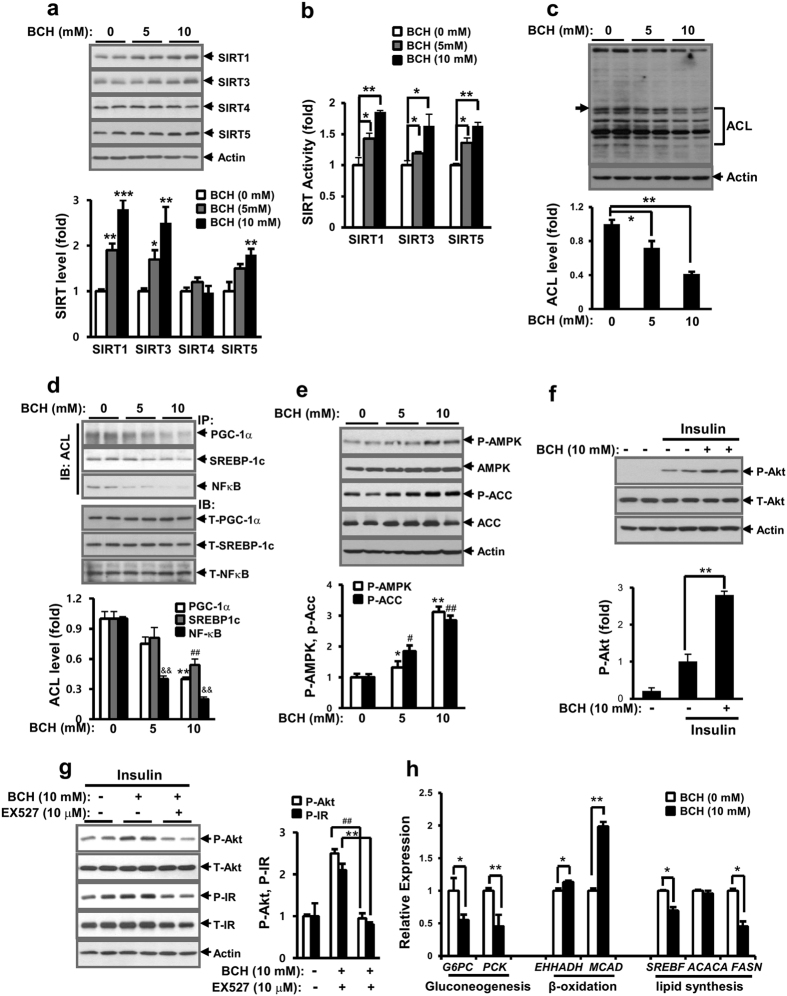
BCH treatment stimulated the activities of SIRTs and AMPK and potentiated insulin-stimulated phospho-Akt in hepatocytes. Primary hepatocytes isolated from normal mice were treated with BCH for 12 h. (**a**) Levels of SIRT1, SIRT3, SIRT4, and SIRT5 were determined by immunoblotting with respective antibodies. (**b**) Enzymatic activities of SIRT1, SIRT3, and SIRT5 were measured using respective assay kits. (**c**) Acetylated proteins in hepatocytes were detected by immunoblotting with anti-acetyl lysine (ACL) antibody. The level of one representative acetylated protein band (arrow) was determined using the one-dimensional Quantity One^®^ image analysis system. (**d**) Deacetylation of SIRT1-targeted proteins was measured by immunoblotting (IB) with anti-ACL antibody after immunoprecipitation (IP) of PGC-1α, SREBP1c, and NF-κB with respective antibodies. (**e**) Levels of phospho-AMPK (P-AMPK) and phospho-ACC (P-ACC) were analyzed by immunoblotting with anti-phospho-AMPK and anti-phospho-ACC antibodies, respectively. (**f**) Hepatocytes isolated from HF/HFr-fed C57BL/6J mice were treated with BCH for 12 h and then, stimulated with insulin for 30 min. Levels of phosphoserine-Akt (P-Akt) and phosphotyrosine-insulin receptor (P-IR) were determined by immunoblotting with anti-P-Akt and anti-P-IR antibodies, respectively. (**g**) Preventive effect of SITR1 inhibitor EX527 on BCH-induced improvement of insulin resistance was measured by immunoblotting analysis of insulin-stimulated P-Akt and P-IR levels. (**h**) Relative expressions of genes related to gluconeogenesis, beta oxidation and lipid synthesis were determined through real time-PCR. Full-length blots are presented in Supplementary Fig. 7. Data were represented as means ± SEM of at least three independent experiments. *p < 0.05, **p < 0.01, ***p < 0.001, ^$$^*p* < 0.01, ^#^*p* < 0.05, ^##^*p* < 0.01 vs. BCH-untreated group.

Next, we determined whether BCH treatment could stimulate the activities of SIRTs and AMPK and potentiate insulin-stimulated signals, because reductive amination produces NAD+ and an elevated NAD+/NADH ratio is associated with the activation of SIRT/AMPK related to restoration of insulin sensitivity^25^,^26^. First, expression levels of cytoplasmic/nuclear SIRT1 and mitochondrial SIRTs such as SIRT3, SIRT4, and SIRT5 were determined by immunoblotting analysis. As shown in Fig. 2a, BCH treatment dose-dependently increased the protein levels of SIRT1, SIRT3, and SIRT5 in primary hepatocytes. In accordance with the enhanced expression of SIRT1, SIRT3 and SIRT5, BCH also increased the activities of SIRT1, SIRT3, and SIRT5 in a dose-dependent manner (Fig. 2b). However, BCH did not influence the expression level of SIRT4 (Fig. 2a). When acetylated proteins in primary hepatocytes were investigated by immunoblotting with anti-acetyl lysine (ACL) antibodies, BCH dose-dependently reduced the amount of acetylated lysine in most cellular proteins in hepatocytes (Fig. 2c). In particular, the acetylated forms of PGC-1α, SREBP-1c, and NFκB, as known target proteins of SIRT1, were significantly reduced by BCH treatment (Fig. 2d). The activity of AMPK was determined by measuring the phospho-form of AMPK and acetyl-CoA carboxylase (ACC). As shown in Fig. 2e, the levels of phospho-AMPK and phospho-ACC were elevated by the BCH treatment in a dose dependent manner. These data suggest that BCH treatment was able to stimulate enzymatic activity of SIRTs/AMPK related to metabolism of fatty acid oxidation and energy production and shift intracellular metabolic milieu to a metabolic state under the conditions of fast or calorie restriction. On the other hand, BCH restored the insulin-stimulated signaling pathway blunted by the HF/HFr-fed diet. The levels of insulin-induced phospho-Akt (P-Akt) in HF/HFr-fed mouse hepatocytes were higher in BCH-treated mouse than those in BCH-untreated (Fig. 2f). In particular, a SIRT1 inhibitor EX527 significantly prevented BCH-induced increased phosphorylation of insulin signaling molecules (Fig. 2g), suggesting that SIRT1 activation was involved in BCH-induced improvement in insulin resistance in HF/HFr-fed mouse hepatocytes. In accordance with the restoring effect of BCH on insulin-stimulated signals, the expression levels of glucose-6-phosphatase (*G6PC*) and phosphoenolpyruvate carboxykinase (*PCK*) were significantly decreased by BCH treatment (Fig. 2h). Since the activation of SIRT1 and AMPK was associated with fatty acid oxidation (FAO), we also determined the expression levels of genes involved in beta oxidation. Levels of enoyl-CoA hydratase/3-hydroxyacyl-CoA dehydrogenase (*EHHADH*) and medium-chain acyl-CoA dehydrogenase (*MCAD*) mRNA were elevated by the BCH treatment. However, the expression levels of lipogenic genes such as sterol regulatory element-binding protein 1-c (*SREBF*) and fatty acid synthase (*FASN*) were reduced by BCH (Fig. 2h).

### Short-term treatment of normal C57BL/6J mouse with BCH reduced glucose production from pyruvate

**Figure 3:**
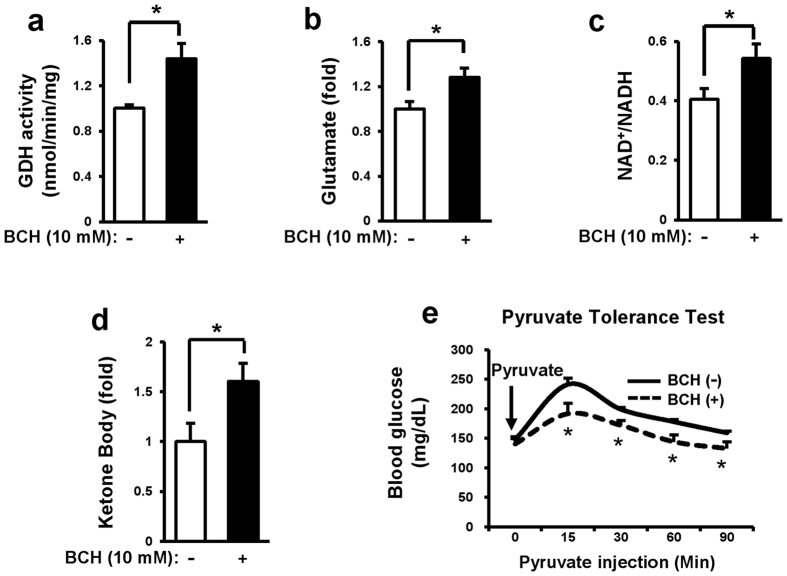
BCH treatment decreased gluconeogenesis but increased ketogenesis in C57BL/6J mice. C57BL/6J mice were challenged with BCH (0.7 g/kg) for 6 h. Soluble fractions were then taken from liver homogenates by differential centrifugation. (**a**) GDH activity, (**b**) level of glutamate, (**c**) NAD+/NADH ratio, and (**d**) level of ketone bodies were measured with corresponding assay kits. (**e**) Mice were treated with 0.7 g/kg BCH for 6 h and then, challenged with pyruvate (2 g/kg). Levels of glucose in blood were measured at 0, 15, 30, and 90 min after pyruvate injection. Data were represented as means ± SEM from two independent experiments (n = 5 per group). *p < 0.05, **p < 0.01 vs. BCH-untreated group.

To determine whether BCH could stimulate reductive amination *in vivo*, non-fasted eight week old C57BL/6J mice were treated with BCH (0.7 g/kg) for 6 h and their glutamate level, NAD+/NADH ratio, and glucose production from pyruvate then were evaluated. Our results revealed that GDH activity, glutamate level, and NAD+/NADH ratio were significantly increased in the liver tissues isolated from BCH-treated mice compared to those in BCH-untreated mice (Fig. 3a, b and c). Furthermore, BCH significantly increased plasma levels of ketone bodies (Fig. 3d), suggesting that BCH treatment induced ketogenic reaction. Serum glucose level was significantly reduced at 15, 30, 60, and 90 min after pyruvate injection in BCH-treated mice (Fig. 3e), implying that BCH treatment reduced pyruvate-induced gluconeogenesis. These results suggested that BCH treatment stimulated reductive amination reaction in non-fasted mouse liver.

### BCH treatment prevented HF/HFr diet-induced fatty liver in C57BL/6J mice

**Figure 4:**
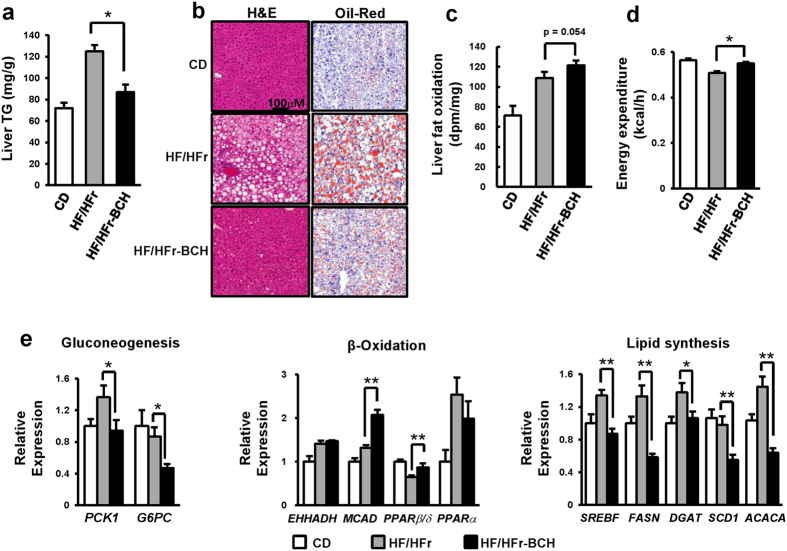
BCH protected HF/HFr-induced fatty liver in C57BL/6J mice. (**a**) Livers were isolated from control diet (CD)-, high fat/high fructose (HF/HFr)-, or BCH-injected high fat/high fructose (HF/HFr-BCH)-fed mice. Liver triacylglycerol (TG) was isolated by Folch extraction method and the level of TG was then measured with a WAKO TG assay kit. (**b**) Liver tissues were stained with hematoxylin and eosin (H&E) or Oil-Red O. (**c**) Fat oxidation was determined through *ex vivo* oxidation assay (Supplementary Methods). (**d**) Energy expenditure was assessed by a comprehensive animal metabolic monitoring system (CLAMS). Metabolic parameters were measured over a 48 h period. The profile of average energy expenditure for each group was obtained every hour during a 24 h course. (**e**) Relative expressions of genes related to gluconeogenesis, beta-oxidation and lipid synthesis were determined through real time-PCR. Data were represented as means ± SEM of ten mice per group (n = 10 per group). *p < 0.05, **p < 0.01 vs. BCH-untreated HF/HFr group.

The preventive effect of BCH on HF/HFr-induced fatty liver was evaluated in C57BL/6J mice. Fatty liver was induced by feeding mice with HF/HFr diet for eight weeks. The effect of BCH on fatty liver was investigated by peritoneal injection of 0.7 g/kg BCH every other day for the same 8 weeks. As shown in Fig. 4a, HF/HFr diet increased liver TG. However, BCH treatment reduced the level of liver TG in HF/HFr-fed mice by 30% (Fig. 4a). In accordance with BCH-induced TG reduction, Oil-Red O fat staining as well as hematoxylin and eosin (H&E) staining clearly demonstrated that BCH prevented HF/HFr-induced fat accumulation in the liver (Fig. 4b). Oxidation experiment with labeled oleic acid demonstrated that HF/HFr diet increased FAO and that BCH treatment further augmented such increase of FAO (Fig. 4c). On the other hand, HF/HFr diet reduced energy expenditure. BCH treatment significantly restored HF/HFr-induced reduction in energy expenditure (Fig. 4d). In accordance with the reduction of TG and increase of FAO in liver tissues, BCH up-regulated mRNA levels of beta-oxidation-related genes while down-regulated mRNA levels of lipogenic genes. Real time-PCR results revealed that BCH significantly increased mRNA level of *MCAD* and peroxisome proliferator-activated receptor (*PPAR) β/δ* but decreased the mRNA levels of *SREBF, FASN*, diacylglyceride acyl transferase (*DGAT*), stearoyl-CoA desaturase (*SCD*) and acetyl-CoA carboxylase alpha (*ACACA*) (Fig. 4e). The expression levels of gluconeogenic genes such as *PCK* and *G6PC* were also down-regulated by the BCH treatment (Fig. 4e).

### BCH treatment significantly reduced the HF/HFr-induced up-regulation of stress/inflammatory signals in C57BL/6J mice

**Figure 5:**
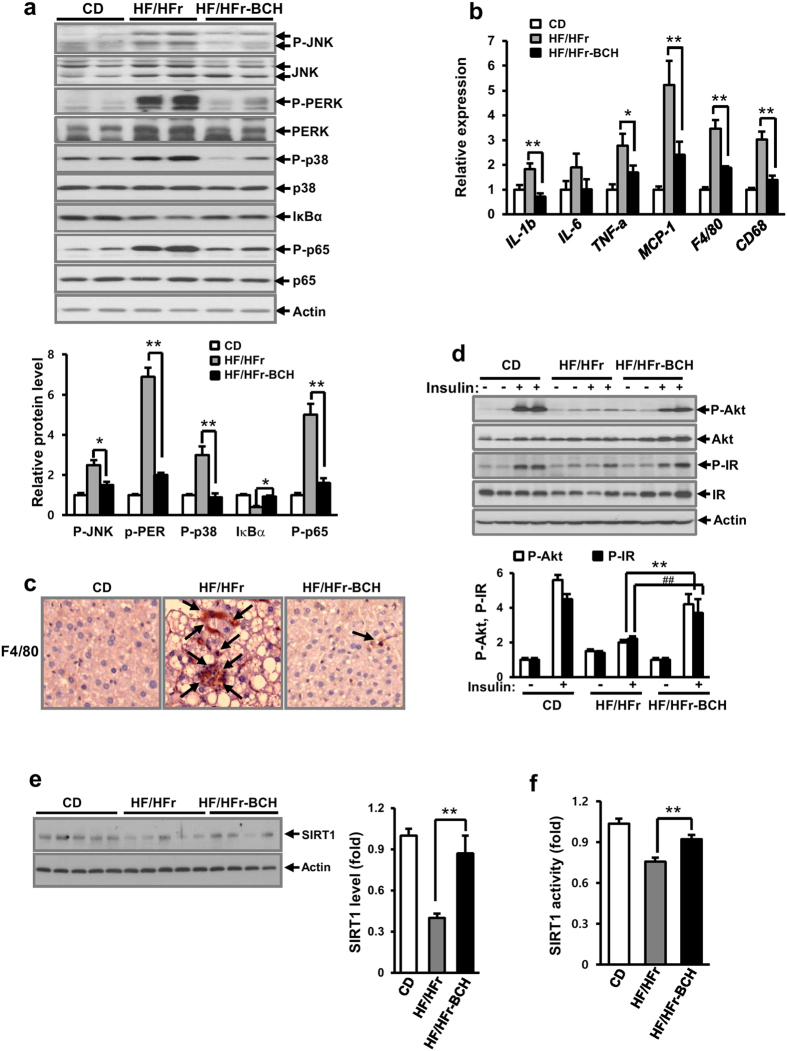
BCH protected HF/HFr-induced hepatic insulin resistance in C57BL/6J mice. (**a**) Liver proteins were extracted with RIPA buffer. Levels of signaling molecules related to ER stress and inflammation were analyzed by immunoblotting with the appropriate antibodies. (**b**) Relative expression of genes related to inflammation was determined through real time-PCR. (**c**) Macrophages in liver tissues were stained with anti-F4/80 antibodies. Arrows designated stained macrophages. (**d**) Levels of P-Akt and P-IR were analyzed by immunoblotting with anti-P-Akt and anti-P-IR antibodies. (**e**) SIRT1 level were measured by immunoblotting with anti-SIRT1 antibody. SIRT 1 activity was determined by SIRT1 activity assay kit. Full-length blots are presented in Supplementary Fig. 8. Data were represented as means ± SEM of eight mice per group. *p < 0.05, **p < 0.01, ^##^*p* < 0.01 vs. BCH-untreated HF/HFr group.

Many stress/inflammation-related signals such as C-Jun N-terminal kinase (JNK), p38, pancreatic endoplasmic reticulum kinase (PERK), and nuclear factor κB (NFκB) have been reported to be involved in the induction of western diet-induced hepatic insulin resistance and liver injury^27^,^28^. To determine whether BCH reduced HF/HFr diet-induced activation of stress/inflammation-related signals, the levels of various signal proteins extracted from 12 week BCH-injected mouse livers were investigated by immunoblotting. As shown in Fig. 5a, the levels of phospho-JNK, phospho-PERK, phospho-p38, and phospho-p65 of NFκB were increased by the HF/HFr diet. However, BCH treatment significantly reduced the HF/HFr-induced up-regulation of stress/inflammatory signals. In addition, HF/HFr diet increased the mRNA level of inflammation-related genes such as *IL-1β, IL-6, TNF-α*, MCP-1, *F4/80*, and *CD68* but BCH significantly restored the HG/HFr-induced up-regulation of inflammation-related genes (Fig. 5b). Furthermore, BCH restored hepatic macrophages increased by HF/HFr diet (Fig. 5c). In accordance with HF/HFr diet-induced up-regulation of stress/inflammation signals, the levels of insulin-stimulated signaling molecules such as phospho-Akt (P-Akt) and phospho-insulin receptor (P-IR) were reduced by the HF/HFr-diet. The BCH also restored the HF/HFr-induced reduction of insulin-stimulated signals (Fig. 5d). To determine whether BCH increased the protein level and enzymatic activity of SIRT1 in HF/HFr-fed mice, SIRT1 level and activity were measured in BCH-treated and HF/HFr-fed mouse liver extracts. As shown in Fig. 5e and 5f, both level and activity of SIRT1 were lower in the HF/HFr-fed mouse liver than those in the control diet (CD)-fed mouse liver. However, BCH treatment significantly restored the HF/HFr-induced reduction in the SIRT1 level and activity.

### BCH ameliorated HF/HFr-induced hepatic injury in C57BL/6J mice

**Figure 6:**
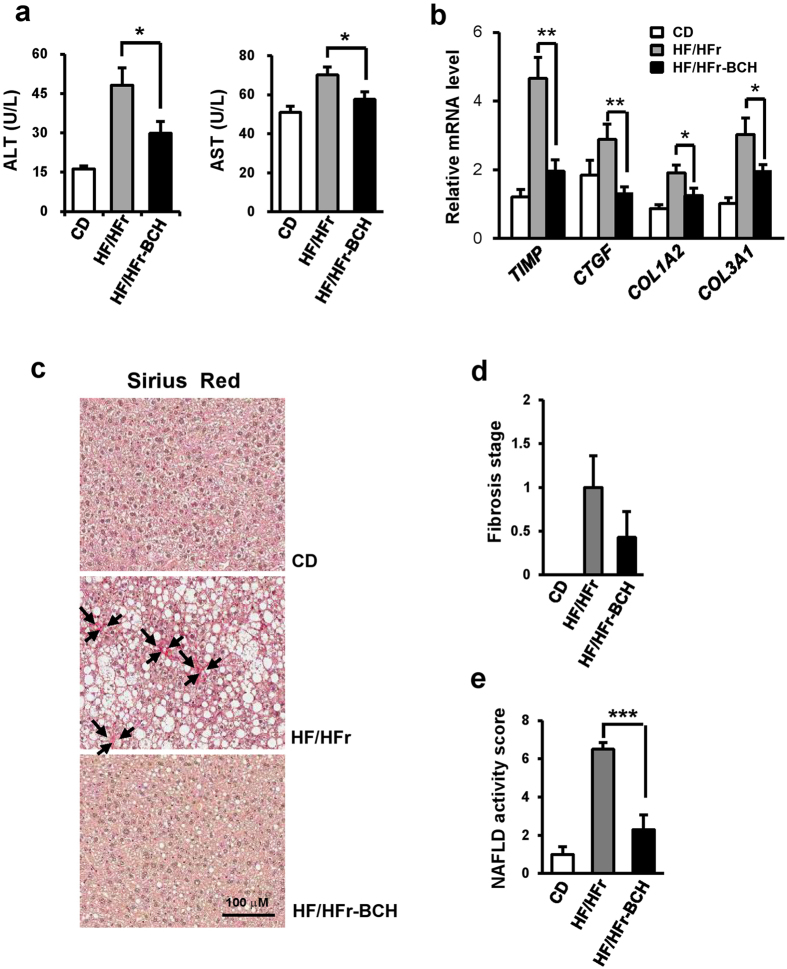
BCH protected HF/HFr-induced hepatic injury in C57BL/6J mice. (**a**) Levels of alanine transaminase (ALT) and aspartate transaminase (AST) in plasma were measured by using an autochemical analyzer. (**b**) Relative expressions of genes related to liver fibrosis were investigated through real time-PCR (**c**) Paraffin sections of liver tissue were stained with Sirius Red Solution. Arrows designated stained collagen. (**d**) Fibrosis stage and (**e**) NAFLD activity score (NAS) (steatosis/inflammation/ballooning degeneration) were determined using the clinical criteria. Data were represented as means ± SEM of eight mice per group. *p < 0.05, **p < 0.01 vs. BCH-untreated HF/Hfr group.

HF/HFr diet for 12 weeks in C57BL/6J mice increased plasma levels of alanine transaminase (ALT) and aspartate transaminase (AST), both of which are markers of hepatocellular injury (Fig. 6a). BCH treatment significantly prevented HF/HFr-induced elevation in ALT and AST levels (Fig. 6a). HF/HFr diet raised the expression levels of fibrosis-related genes such as tissue inhibitor of metalloproteinase (*TIMP*), connective tissue growth factor (*CTGF*), collagen alpha-2(I) chain (*COL1A2*) and collagen alpha-1(III) chain (*COL3A1*) in liver tissues. Furthermore, it increased collagen stained by sirius red in the same tissues. BCH reduced mRNA levels of all liver fibrosis-related molecules and collagen in HF/HFr-fed mouse livers (Fig. 6b and c). In accordance with down-regulation of collagen, BCH tended to reduce fibrosis stage that had been increased by HF/HFr diet, even if the reduction was not statistically significant (Fig. 6d). On the other hand, NAFLD activity score (NAS) that had been increased by HF/HFr diet was significantly reduced by BCH treatment (Fig. 6e). Collectively, BCH treatment was able to prevent HF/HFr diet-induced increases in markers of hepatic fibrosis and injury.

### BCH treatment improved HF/HFr diet-induced hyperglycemia in C57BL/6J mice

**Figure 7:**
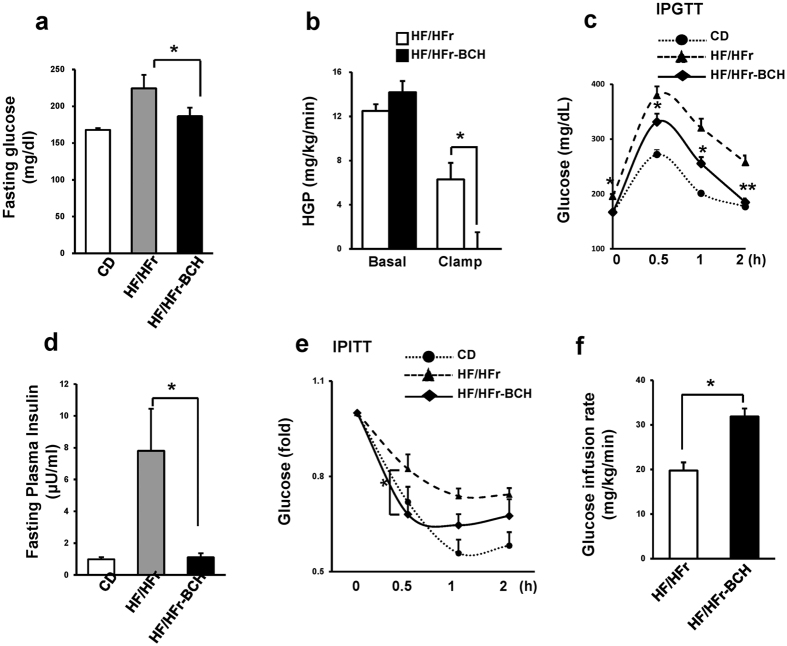
BCH ameliorated HF/HFr-induced hyperglycemia in C57BL/6J mice. (**a**) Blood glucose after fasting for 6 h was measured using an Accu-Check glucometer. (**b**) Hepatic glucose production (HGP) was determined during hyperinsulinemic–euglycemic clamps. (**c**) Intraperitoneal glucose tolerance test (IPGTT) was carried out by measuring glucose levels at 0, 0.5, 1, and 2 h after glucose injection (1 g/kg). (**d**) Plasma insulin level after fasting for 6 h was measured using an insulin radioimmunoassay (RIA) kit. (**e**) Intraperitoneal insulin tolerance test (IPITT) was carried out by measuring glucose levels at 0, 0.5, 1, and 2 h after insulin injection (0.5 U/kg). (**f**) Glucose infusion rates were measured during hyperinsulinemic–euglycemic clamps. Data were represented as means ± SEM of three independent experiments (n = 10 per group). *p < 0.05, **p < 0.01 vs. BCH-untreated Hf/Hfr group.

Since hepatic insulin resistance associated with NAFLD is a causing factor of T2D, we studied whether BCH treatment could prevent HF/HFr diet-induced hyperglycemia. As shown in Fig. 7a, the HF/HFr diet increased fasting plasma glucose level around 43% compared to control diet. The BCH treatment restored HF/HFr-induced increase of fasting glucose near to glucose level of control diet. Euglycemic hyperinsulinemic clamp demonstrated that hepatic glucose production (HGP) was almost completely suppressed in BCH-injected mice (Fig. 7b), suggesting that BCH-induced improvement of fasting hyperglycemia was mainly due to the suppression of HGP. On the other hand, BCH treatment significantly improved HF/HFr-induced impaired glucose tolerance at all-time points (0.5, 1, and 2 h) (Fig. 7c) and also restored increased level of fasting plasma insulin in HF/HFr-fed mice to that in control diet (Fig. 7d). Furthermore, insulin tolerance test (ITT) showed that the slope of glucose reduction in BCH-treated mice at 0.5 h was steeper than that of BCH-untreated HF/HFr-fed mice, supporting that the BCH treatment improved insulin resistance (Fig. 7e). The improvement in glucose infusion rate by the BCH treatment also suggested that BCH improved insulin resistance in HF/HFr-fed mice (Fig. 7f and Supplementary Fig. 3). In accordance with the improvement of insulin resistance, the BCH treatment restored islet hyperplasia in HF/HFr-fed-mice (Supplementary Fig. 4).

## Discussion

This study was performed to determine whether GDH activator BCH could reduce DNL and gluconeogenesis and stimulate the activity of SIRTs and AMPK through stimulation of reductive amination, thus ameliorating HF/HFr diet-induced fatty liver and hyperglycemia in C57BL/6J mice. Our result revealed that treatment with BCH reduced DNL and gluconeogenesis but stimulated activities of SIRT1, SIRT3, SIRT5, and AMPK in cultured hepatocytes. Long-term BCH treatment in HF/HFr-fed C57BL/6J mice prevented HF/HFr diet-induced hepatic steatosis, steatohepatitis, and hyperglycemia. Our results suggest that stimulation of reductive amination through GDH activation is an effective strategy to prevent western diet-induced NAFLD and T2D.

In islet beta cells, the oxidative deamination reaction has been preferentially used to stimulate insulin secretion because beta cells are mainly designed for insulin secretion and have a high capacity for TCA cycle turnover, ATP production, and citrate synthesis for insulin secretion^29^,^30^. In accordance with this assumption, our experiment showed that BCH treatment decreased the glutamate level and NAD+/NADH ratio in INS-1 beta cells (Supplementary Fig. 2). While the main function of GDH in the kidney is ammonia production from glutamate through stimulation of oxidative deamination^13^,^31^, it has been reported that GDH can catalyze both oxidative deamination and the reductive amination reaction in the liver^32^. In particular, ammonia that has escaped from urea shunting could be incorporated into glutamate for ammonia detoxification through stimulation of reductive amination in hepatocytes^33^. BCH mainly activated reductive amination in hepatocytes in our experimental conditions, because we cultured the hepatocyte in medium with relatively high energy potential and raised the mice on a high fat/high fructose diet with relatively low protein. This condition having high energy potential may favor the reductive amination reaction in liver because amino acids are not required for energy production; instead ammonia-saving metabolism is necessary. In fact, our studies demonstrated that BCH elevated the glutamate level and NAD+/NADH ratio in liver tissues as well as cultured hepatocytes. Furthermore, it enhanced levels of ketone bodies, suggesting that BCH prefers the reductive amination reaction in liver tissues. Although BCH is known as an inhibitor of L-type amino acid transporter (LAT), BCH does not seem to play an inhibitory role of LAT in our system since LAT was not highly expressed in normal hepatocytes^34^ and mTOR/S6K signal down-regulated through amino acid insufficiency was not reduced by BCH treatment (Supplementary Fig. 5).

In fasting or calorie restriction conditions, oxidative deamination reaction is supposed to be preferentially operated in liver since enhanced gluconeogenesis generally requires TCA cycle intermediates supplied from amino acids^35^. However, in feeding condition, cataplerotic reaction for DNL is likely to be dominated by inhibition of reductive amination since GTP is known as an allosteric inhibitor of GDH^14^. Addition of BCH as an analogue of leucine may stimulate reductive amination even in feeding condition since leucine has been reported to be able to displace GTP from allosteric inhibitor binding sites in GDH and stimulate GDH activity^36^. Collectively, it is believed that BCH treatment dominates the reductive amination under high calorie HF/HFr-feeding condition. Because glutamate produced through reductive amination may be involved in glutamine production through glutamine synthetase, it was expected that BCH treatment would increase the level of glutamine but decrease the ammonia level. As shown in Supplementary Fig. 6, BCH increased the glutamine level but reduced the ammonia level in BCH-treated mouse liver under the feeding condition. In conjunction with the increase of glutamine, BCH significantly increased the glutamine synthetase activity in liver extract but did not influence the glutaminase activity. These data suggest that the GDH activator BCH stimulates glutamine synthesis metabolism as well as glutamate synthesis metabolism in liver when feeding a diet. Although stimulation of reductive amination through BCH treatment improved HF/HFr-induced metabolic alteration, overexpression of GDH may not be a good means to reduce metabolic alterations induced by high calorie western diets since molecular increase of GDH itself does not guarantee a stimulation of reductive amination.

Since fructose in the HF/HFr diet plays a significant role in fat accumulation through increasing DNL in the liver^37^, decreased DNL by the BCH treatment might cause reduced accumulation of liver fat in HF/HFr-fed mice. Furthermore, stimulation of fat oxidation is also likely to be involved in the reduction of liver fat in BCH-treated mice^38^,^39^. In fact, treatment with BCH increased expression and activities of various SIRTs, such as SIRT1, SIRT3 and SIRT5, which are involved in metabolism of FAO and ATP generation. The report that fasting and calorie restriction induced expression of cytosolic/nuclear SIRT1 and mitochondrial SIRT3 and SIRT5 suggests that BCH treatment induce fasting and calorie restriction state in liver^40^. In particular, SIRT1 activation is likely to be an important mediator for BCH-induced improvement in insulin resistance since SIRT1 inhibitor prevented BCH-induced beneficial effect on hepatic insulin resistance in HF/HFr-fed mice. Our data also demonstrated that BCH up-regulated the expression of genes involved in fat oxidation. Enhanced ketogenesis was another indicator of excessive fat oxidation by the BCH treatment. On the other hand, BCH improved hyperglycemia in HF/HFr-fed mice. The improvement of fasting hyperglycemia might be caused by lowered production of hepatic glucose in BCH-treated mice. In fact, BCH was able to reduce glucose production from pyruvate in hepatocytes. The reduction of hepatic glucose production could be due to preferential use of TCA cycle intermediates for glutamate synthesis in BCH-treated mice rather than for gluconeogenesis. It is also possible that BCH-induced improvement of insulin sensitivity plays a role in insulin-stimulated suppression of gluconeogenic gene expression.

In accordance with BCH-induced restoration of insulin signals, stress/inflammation signals that play key roles in the induction of insulin resistance in HF/HFr-fed mouse liver were significantly down-regulated by the BCH treatment. The levels of ER stress signals such as phospho-PERK and phospho-JNK as well as inflammatory signal phospho-p65 were almost completely normalized by the BCH treatment. HF/HFr-induced increases in mRNA levels of IL-1β and TNF-α were restored by the BCH treatment. F4/80-positive macrophages in HF/HFr-fed mouse liver tissues were also reduced in BCH-treated mouse livers. BCH was also able to protect against HF/HFr-induced hepatic injury and fibrosis. All these data suggest that stress/inflammatory/toxic signals activated by long-term HF/HFr diet can be prevented by stimulating reductive amination reaction. Since there are cross-talks among metabolic organs such as the liver, adipose, and muscle tissue^41^,^42^, it is possible that improvement of liver metabolism by BCH treatment can improve systemic insulin resistance in HF/HFr-diet mice. Reduction of plasma insulin level, improvement of insulin tolerance, and increase of glucose infusion rate supported systemic improvement of insulin resistance by BCH treatment in HF/HFr-fed mice.

The reasons why continued intake of HF/HFr diet activated stress/inflammation signals and led to insulin resistance and hepatic injury were not clearly determined. Elevated fatty acid concomitant with hyperglycemia and hyperinsulinemia has been thought to play a role in the induction of hepatic inflammation and insulin resistance^43^. Mitochondrial dysfunction has been implicated the activation of stress/inflammation signals through producing reactive oxygen species (ROS)^44^,^45^. While dysfunction in mitochondrial oxidation might play a role in the induction of hepatic inflammation, enhanced mitochondrial FAO without concomitant up-regulation of the mitochondrial respiratory chain activity has been suggested to be a cause of hepatic inflammation^46^,^47^,^48^. In particular, Koliaki *et al*. have proposed that increased β-oxidation is an adaptive response to lipid oversupply in states of steatosis that can be followed by progressive decline in liver mitochondrial functions^49^. Long-lasting decrease in NAD+/NADH ratio possibly due to enhanced FAO with insufficient mitochondrial respiratory activity may have contributed to the development of metabolic disorders through excessive production of ROS^24^,^50^. Reports that reduction in NAD+/NADH ratio is involved in insufficient activation of SIRT/AMPK support the hypothesis that a low level of NAD+/NADH ratio contributes to the induction of stress/inflammation signals through ROS^23^,^51^. Supporting this hypothesis, our data revealed that BCH treatment could increase NAD+/NADH ratio and SIRT1/AMPK activity and improve most metabolic disorders by HF/HFr western diet.

In conclusion, GDH activator BCH prevented most metabolic alterations caused by HF/HFr diets, possibly through stimulation of reductive amination. Activation of SIRTs/AMPK through increasing NAD+/NADH ratio and reduction of cataplerosis for DNL and gluconeogenesis might play roles in the protective effect of BCH on HF/HFr-induced hepatic steatohepatitis and hyperglycemia. Since metabolic milieu through enhanced reductive amination mimics metabolic conditions in fasting and calorie restriction states, i.e, enhanced FAO, reduced lipogenesis, and enhanced ketogenesis, with the exception of enhanced gluconeogenesis, stimulation of reductive amination through GDH activation can be used as a new strategy to prevent western diet-induced NAFLD and T2D.

## Methods

### Materials

Most chemicals including 2-aminobicyclo-(2,2,1)-heptane-2-carboxylic acid (BCH) were obtained from Sigma Aldrich (St. Louis, MO). Radio-labeled glucose and oleate were purchased from Perkin-Elmer NEN (Waltham, MA). Anti-actin, SIRT1, and JNK antibodies were obtained from Santa Cruz Biotechnology (Santa Cruz, CA). Anti-SIRT3, SIRT5, Akt, phospho-Akt, AMPK, phospho-AMPK, ACC, phospho-ACC, phospho-JNK, insulin receptor, phospho-insulin receptor, acetylated lysine, PERK, phospho-PERK, Bip, p-38, phospho-p38, p65, phospho-p65, and IκBα antibodies were purchased from Cell Signaling Technology (Beverly, CA). Anti-F4/80 antibody was obtained from eBioscience (San Diego, CA). Anti-SIRT4 antibody was obtained from Abcam (Cambridge, UK).

### Isolation of hepatocytes

Mice were given a single intraperitoneal injection of 50 mg/kg sodium pentobarbital to achieve deep anesthesia. Caudal vena cava was cannulated and the portal vein was cut. The liver was perfused initially with 50 ml of 37 °C sterile calcium- and magnesium-free Hank’s Balanced Salt Solution (HBSS) supplemented with 0.5 mM EGTA, 5.5 mM glucose, and penicillin/streptomycin (P/S) followed by 40 mL of 30 °C sterile HBSS supplemented with 1.5 mM calcium chloride, 5.5 mM glucose, P/S, and 0.39 g Type IV collagenase (920 CDU/mg, Sigma). Livers were then surgically removed and gently agitated with William’s medium E containing 1% fetal bovine serum (FBS, Sigma) and P/S. The solution was passed through sterile gauze to separate undigested liver. The medium containing digested liver was adjusted to 50 ml with FBS-containing medium and subjected to centrifugation at 50 g for 2 minutes. The supernatant was aspirated and cell pellets were then washed three times with 50 ml of FBS-free medium. Hepatocyte viability was evaluated by trypan blue exclusion. Viability of >85% was set as the minimum criterion for hepatocyte utilization.

### Animal treatment

Six-week-old male C57BL/6J mice were purchased from Japan SLC (Shizuoka, Japan) and housed in a temperature-controlled room (22.8 °C) with 12 h of light. Following 2 weeks of adaptation, 8-week-old mice were randomly assigned into the following three groups (8–10 mice per group): 1) saline-injected control diet (CD) group; 2) saline-injected high fat/high fructose (HF/HFr) diet group; 3) BCH-injected HF/HFr (HF/HFr-BCH) diet group. For the control group, mice were fed normal chow diet containing 10% fat (D12450B; Research Diets Inc., New Brunswick, NJ) and water. For the HF/HFr group, mice were fed chow diet with 60% fat (D12492; Research Diets Inc.) and 30% fructose water. For the HF/HFr-BCH group, BCH (0.7 g/kg) was injected intraperitoneally every other day. All animal care and treatments were conducted according to Ajou Institutional Animal Care guidelines. All experimental protocol for animal study were approved by Ajou Institutional Animal Care Committee (Permission number: AMC126).

### Hyperinsulinemic-euglycemic clamp

After an overnight fast, [^3^H] glucose was infused at a rate of 0.05 μCi/min for 2 h. Following a basal period, hyperinsulinemic-euglycemic clamp was performed for 140 min with a primed/continuous infusion of human insulin (126 pmol/kg during priming and 18 pmol/kg per min during infusion). Plasma glucose was maintained at basal concentration (~6.7 mM) by infusion of glucose at variable rates. Glucose infusion rate (GFR) was determined during clamp. To estimate whole body glucose turnover rate during clamp, [^3^H] glucose was infused at a rate of 0.1 μCi/min. Basal hepatic glucose production (HGP) was measured as a ratio of basal [^3^H] glucose infusion rate (dpm/min) to the specific activity of plasma glucose (dpm/μmol) in plasma at the end of the basal period. The clamp HGP in insulin-stimulated condition was measured by subtracting GFR from whole body glucose turnover rate^18^.

### Tolerance tests of glucose, insulin, and pyruvate

For glucose and pyruvate tolerance tests, glucose (1 g/kg) and pyruvate (2 g/kg) were administered, respectively via injection into the peritoneal cavity after a 6-h fast. For insulin tolerance test, a peritoneal injection of insulin (0.5U/kg) was carried out to mice fasted for 6 h. Blood glucose was taken from tails. Glucose concentration was then determined using an Accu-Check glucometer (Roche, Mannheim, Germany).

### Immunoblotting

RIPA buffer (150 mM NaCl, 1% NP-40, 0.5% deoxycholate, 0.1% sodium dodecyl sulfate, 50 mM Tris.HCl at pH 7.5, protease inhibitor cocktail; Roche Applied Science, Mannheim, Germany) was used to extract cellular proteins. Protein lysates were obtained by differential centrifugation (10,000x g, 10 min). Protein concentrations in lysates were determined using protein assay kits (Bio-Rad, Hercules, CA). An equal volume of 2 x SDS sample buffers [125 mM Tris.Cl, (pH 6.8), 4% SDS, 4% 2-mercaptoethanol, 20% glycerol] was added to cell lysates. Equivalent amounts of protein (30 μg) were loaded onto 10–15% polyacrylamide gels, fractionated through electrophoresis, and transferred onto polyvinylidene fluoride (PVDF) membranes (Millipore, Bedford, MA). Membranes were sequentially incubated with primary and secondary antibodies in Tris-buffered saline containing 0.05% Tween-20 (TBST) supplemented with 5% (w/v) non-fat dry milk. Immunoreactive bands were developed using enhanced chemiluminescence detection system (Amersham Pharmacia Biotech, Arlington Height, IL). Band intensity was determined by densitometric analysis using a one-dimensional Quantity One^®^ 1D image analysis system (Bio-Rad, Hercules, CA).

### Real Time-Polymerase Chain Reaction

Total RNAs were extracted with TRIzol (Invitrogen, Carlsbad, CA). RNA quantity and purity were determined with a NanoDrop 1000 spectrophotometer (Thermo Fisher Scientific Waltham, MA). cDNAs were synthesized using Takara AMV reverse transcriptase (Otsu, Shiga, Japan) according to the manufacturer’s instructions. Quantitative real-time PCR was performed with TAKARA SYBR Premix ExTaq and individually synthesized primers using Thermo Cycler Dice TP850 (Takara, Otsu, Shiga, Japan). Sequences of primer sets were optimized using Primer 3 program and are listed in Supplementary Table 1. All genes were amplified at 95 °C for 5 sec and 60 °C for 30 sec for denaturation and annealing/polymerization, respectively. Relative quantity of amplified DNAs was analyzed using software supplied from TP850.

### Statistical analysis

Data were presented as means ± SEM. Statistical significance of difference between two groups was analyzed using unpaired Student’s *t*-test. Energy expenditure was adjusted for body weight by analysis of covariance (ANCOVA). Statistical analyses were performed using SPSS software version 13.0 (SPSS, Inc., Chicago, IL, USA). Statistically significant differences were considered when P values were less than 0.05.

Methods measuring the levels of various biomolecules (glutamate, NAD+, NADH, ketone body, glucose, insulin, alanine transaminase, and aspartate transaminase), GDH activity, SIRT1 activity, DNL, liver fat, glucose production, energy expenditure, and fatty acid oxidation are included in Supplementary Methods. Methods for INS-1 cell culture and all histological analysis are also included in Supplementary Methods.

## Additional Information

**How to cite this article**: Han, S. J. *et al*. Glutamate dehydrogenase activator BCH stimulating reductive amination prevents high fat/high fructose diet-induced steatohepatitis and hyperglycemia in C57BL/6J mice. *Sci. Rep.* **6**, 37468; doi: 10.1038/srep37468 (2016).

**Publisher's note:** Springer Nature remains neutral with regard to jurisdictional claims in published maps and institutional affiliations.

## Supplementary Material

Supplementary Information
