# Supplementary material for: Glutamate dehydrogenase activator BCH stimulating reductive amination prevents high fat/high fructose diet-induced steatohepatitis and hyperglycemia in C57BL/6J mice
Source: Sci Rep. 2016 Nov 22;5:37468. doi: 10.1038/srep37468 (PMC5118703; doi:10.1038/srep37468)
Supplement: Supplementary Information [file srep37468-s1.pdf]

Supplementary Information

Glutamate dehydrogenase activator BCH stimulating reductive amination prevents high fat/high fructose diet-induced steatohepatitis and hyperglycemia in C57BL/6J mice

Seung Jin Han, Sung-E Choi, Sang-A Rhee, Jong Gab Jung, Ik-Rak Jung, Maureen Shin, Seok Kang, Hyunhee Oh, Hae Jin Kim, Dae Jung Kim, Ji Eun Kwon, Cheol Soo Choi, Kwan Woo Lee, Yup Kang

Supplementary Methods

Measurement of GDH activity. Hepatocytes or liver tissues were homogenized in extract buffer supplied in Biovision GDH activity assay kit (#K729-100, Milpitas, CA, USA). GDH activity in soluble fraction obtained after centrifugation (10,000g, 10 min) was determined using a colorimetric assay according to the manufacturer's protocol.

Measurement of glutamate. Glutamate assay kit (Biovision #K629-100) was used to measure glutamate levels according to the manufacturer's protocol.

Measurement of NAD+ and NADH. Cells or tissues were homogenized in cell extract buffer supplied in Biovision NAD+ and NADH assay Kit (#K808-200 and #K337-100, respectively). NAD+ and NADH in the soluble fraction obtained by centrifugation (10,000g, 10 min) were measured by enzymatic reactions according to the manufacturer's instruction.

Measurement of ketone bodies. Colorimetric assay kit purchased from Biovision (#K632-100) was used to measure ketone bodies according to the manufacturer's protocol.

Biochemical analysis for blood samples. Blood obtained from mice was immediately centrifuged at 13,000 rpm for 1 min at 4 ^{o}C. Upper plasma was collected and stored at -80 ^{o}C. Plasma glucose levels were measured using glucose oxidase method. Plasma insulin levels were determined using an insulin RIA kit (Linco Research, Billerica, MA). Plasma levels of triglyceride, total cholesterol, alanine aminotransferase (ALT), and aspartate aminotransferase

(AST) were measured using an autochemical analyzer (Cobas c111, Roche, Germany).

Measurement of SIRT activity. Deacetylase activities of SIRT1, SIRT3 and SIRT5 were determined by measuring fluorescence released from different peptides supplied from kits. Fluorescence-conjugated peptides comprised of parts of human p53, histone, and carbamoyl phosphate synthetase 1were used for measuring SIRT1 (Sigma Aldrich, St. Louis, MO), SIRT3 (Abcam, Cambridge, UK), and SIRT5 (Abcam) activities, respectively.

Measurement of glucose incorporation into lipid. *De novo* lipogenesis was determined by measuring radio-labeled glucose (^{14}C-glucose) accumulated in triacylglycerol (TG) in hepatocytes. Briefly, cells ($1x10^6$) incubated with ^{14}C-glucose (0.1 μCi/ml, PerkinElmer) for 3 h were collected by differential centrifugation (500g, 5 min). Cell pellets were suspended in 1 ml of chloroform: methanol (2:1) mixture. Following an addition of 0.3 ml of water, lipids in the organic phase were separated by differential centrifugation (4,000g, 10 min) and saved in a new tube. The radioactivity in the lipid fraction was determined using a scintillation counter (Tri-Carb 2100TR, Packard, USA).

Measurement of liver triacylglyceride. Tissue triglyceride was extracted using Folch extraction method (*J Biol Chem* **226,** 497–509. 1957) and measured with commercially available kit (LabAssay™ triglyceride, Wako Diagnostics, Japan) using N-ethyl-N-(2-hydroxy-3-sulfopropyl)-3,5-dimethoxyaniline sodium salt (DAOS).

Glucose production assay in primary hepatocytes. Hepatocytes were cultured in glucose-free DMEM supplemented with 20 mM lactate and 2 mM pyruvate for 2 h. Glucose concentration in the media was measured by using an Accu-Check glucometer (Roche, Mannheim, Germany).

Ex Vivo fat oxidation in liver tissues. Livers were quickly removed from mice and placed in 25-ml flasks fitted with center wells containing 1 N NaOH and filter paper strip to trap $^{14}CO_2$. Flasks were capped with bottle stoppers. The incubation media contained 3 ml of Krebs-Ringer phosphate buffer, 2 μCi of [1-^{14}C] oleic acid and cold oleic acid (0.6 mM final concentration) in complex with BSA. Tissues were incubated in a 37 °C shaking water bath for 30 min. One ml of 0.5 N sulfuric acid was injected into the media to release $^{14}CO_2$. Flasks were maintained at 50 °C for 3 h to transfer $^{14}CO_2$ to NaOH in the center well. After acid treatment, contents in the center well were transferred to scintillation fluid and [^{14}C] radioactivity was measured.

Measurement of energy expenditure. Energy expenditure was assessed with a metabolic monitoring system (comprehensive animal metabolic monitoring system, CLAMS: Columbus Instruments) for 4 days for each mouse after 2 days of acclimation followed by 2 days of measurement as described previously (*Proc Natl Acad Sci USA* **104**, 16480-16485. 2007). Respiratory quotient (RQ) was calculated from gas exchange data. RQ was the ratio of VCO_2 to VO_2. Energy expenditure (EE) was calculated with the following formula: EE = (3.815 + 1.232 x RQ) x VO_2. Hourly energy expenditure and energy intake_values were averaged for the 24 h period.

Culture of INS-1 beta cells. INS-1 rat insulinoma cells kindly supplied by Dr. Wollheim (University of Geneva Medical Center, Geneva, Switzerland) were grown in RPMI 1640 medium supplemented with 10% fetal bovine serum (Invitrogen), 100 U/mL penicillin, 100 g/mL streptomycin, and 10 mM HEPES at 37 ℃ in a humidified atmosphere containing 95% air and 5% CO_2.

Oil Red O staining. Liver tissue frozen in OCT compound were cut at 5 μm thickness, mounted onto slides, fixed with 10% formalin, and stained in 0.5% Oil Red O solution. Red oil drops were observed under light microscope.

F4/80 staining. Frozen tissues sections were blocked with 5% normal bovine serum. Antigens were reacted with anti-mouse F4/80 primary antibody and horse radish peroxidase (HRP)-conjugated rabbit anti-rat IgG secondary antibody. Brown colors were detected using 3,3'-diaminobenzidine (DAB) as HRP substrate.

Sirius Red staining. Paraffin sections were de-waxed and hydrated with phosphate-buffered saline. Collagens were stained in Sirius Red solution (Abcam) for one hour. After washing with acidified water, tissues were dehydrated with 100% ethanol. Nuclei were stained with hematoxylin.

Histological assessment. Liver tissues fixed with phosphate buffered saline containing 4% paraformaldehyde were embedded in paraffin, sliced (4 μm sections), mounted onto slides,

and stained with hematoxylin and eosin (H&E) according to standard procedures. Histological assessment was performed by a pathologist blinded to the study. The fibrosis stage and NAFLD activity score (NAS) (steatosis/inflammation/ballooning degeneration) were determined using the clinical criteria outlined by Kleiner et al (*Hepatology* 41, 1313-1321. 2005).

Supplementary Table

Supplementary Table 1. Primer sequences used for quantitative real time-PCR

Gene	GenBank Accession No	Forward (5'-3')<br>Reverse (5'-3')	Amplicon size
ACACA	NM_1333360.2	ATCGACACTGGCTGGCTGGA<br>GCCCCACACACAACTCCCAA	86
CD68	NM_001291058.1	AGGGACACTTCGGGCCATGT<br>GGGTGATGCAGAAGGCGATG	120
DGAT	NM_010046.2	TGGTGTGTGGTGATGCTGATC<br>GCCAGGCGCTTCTCAA	194
EHHADH	NM_023737.3	AAGCCGGGAGCCTTTCTGTG<br>CATGATGTGGGCTGGGGAGA	120
F4/80	NM_010130.4	CCCGTGTTGTTGGTGGCACT<br>GCTTTGGCTGGATGTGCTGG	80
FASN	NM_007988.3	CCTGGATAGCATTCCGAACCT<br>AGCACATCTCGAAGGCTACACA	122
G6PC	NM_008061.3	AGATGGTGTGAGCGGCCAGA<br>CAACCCCAAGAGGGTTCCCA	87
IL-1β	NM_008361.3	TCTCGCAGCAGCACATCAACA<br>CCTGGAAGGTCCACGGGAAA	105
IL-6	NM_001314054.1	CCATCCAGTTGCCTTCTTGGG<br>GCCGTGGTTGTCACCAGCAT	45
MCAD	NM_007382.5	GATCGCAATGGGTGCTTTTGATAGAA<br>AGCTGATTGGCAATGTCTCCAGCAAA	291
MCP-1	NM_011333.3	CAGCCAGATGCAGTTAACGC<br>GCCTACTCATTGGGATCATCTTG	73
PCK1	NM_011044.2	GACACAGTGCCCATCCCCAA<br>TGGGAACCTGGCGTTGAATG	93
PPARα	NM_001113418.1	AGAAGTTGCAGGAGGGGATT<br>TTGAAGGAGCTTTGGGAAGA	92
PPARβ/δ	NM_011145.3	AATGCGCTGGAGCTCGATGAC<br>ACTGGCTGTCAGGGTGGTTG	166
RPL32	NM_172086.2	AAGCGAAACTGGCGGAAACC<br>CCCATAACCGATGTTGGGCA	90
SCD1	NM_009127.4	CACACGCCGACCCTCACAAT<br>TTTGACAGCCGGGTGTTTGC	86
SREBF	NM_0011480.4	GCTGTTGGCATCCTGCTATC<br>AGCTGGAAGTGACGGTGGT	174
TNF-α	NM_013693.2	AGCCCCCAGTCTGTATCCTT<br>GGTCACTGTCCCAGCATCTT	113
TIMP	NM_001044384.1	TCCCCAGAAATCAACGAGAC<br>CATTTCCCACAGCCTTGAAT	88
CTGF	NM_010217.2	CTTTCTGGCTGCACCAGTGT<br>GGCAGAGTGGTGGTTCTGTG	102
COL1A2	NM_007743.3	CTGGGTGAACCTGGTCCTCT<br>ACCAGGGGCACCATTAACTC	100
COL3A1	NM_009930.2	GTCCAAAGGGTGAAGTCGGT<br>CAGCTCCACCTCTAGCACCA	125

Supplementary Figures

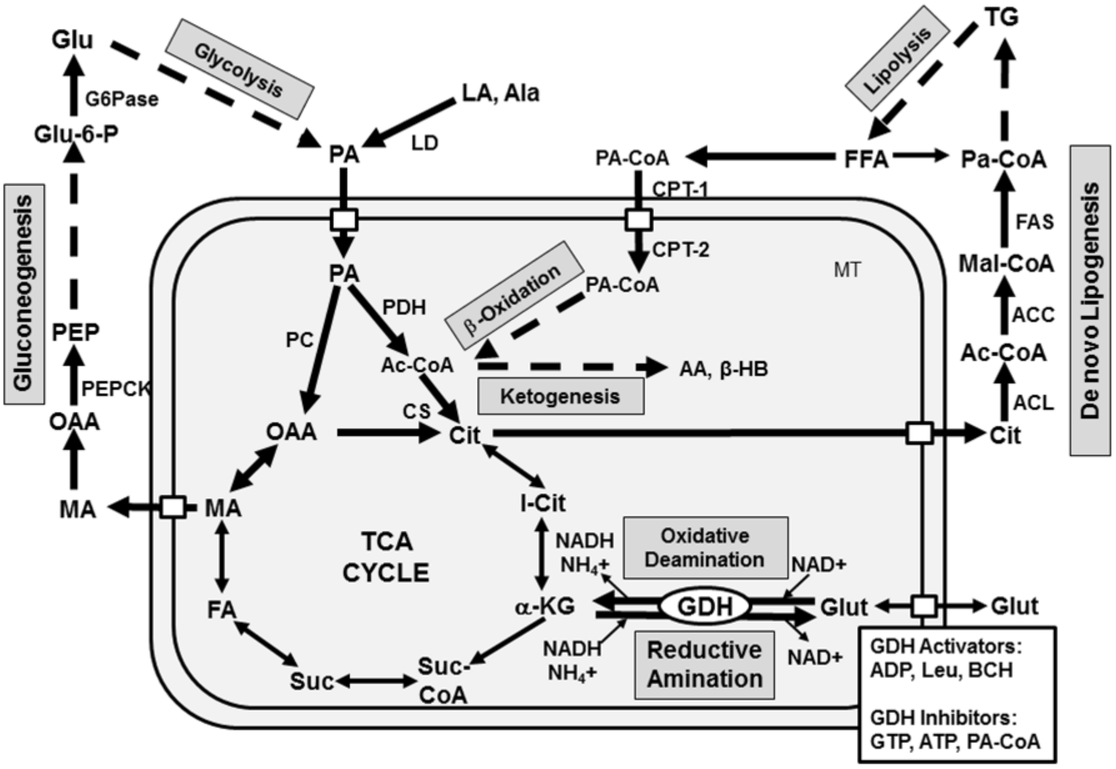

Supplementary Fig. 1. Tricarboxylic acid cycle as a metabolic crossroad for oxidation and synthesis of glucose, fatty acid, and amino acids. While glycolysis and gluconeogenesis describe the metabolism of oxidation and synthesis of glucose, beta-oxidation and de novo lipogenesis (DNL) describe the oxidation and synthesis of fatty acid. Ketogenesis is an alternative metabolism of fatty acid oxidation in conditions with low TCA cycle intermediate pools. On the other hand, oxidative deamination and reductive amination represent the metabolism of oxidation and synthesis of amino acids. Glutamate undergoes oxidative deamination for the production of alpha-ketoglutarate, ammonia, and NADH through the activation of glutamate dehydrogenase (GDH) in a fasting condition. In a feeding

condition, glutamate is presumed to be synthesized from alpha-ketoglutarate and ammonia through reductive amination using the same enzyme, concomitantly releasing NAD+. However, large amounts of GTP synthesized in highly respiratory conditions may block the reductive amination reaction due to its inhibitory action for GDH. Thus, TCA intermediates preferably siphon into DNL. BCH, an analogue of leucine, can stimulate the reductive amination reaction for the production of glutamate and NAD+ in a feeding condition since it has been reported that leucine could displace GTP from the allosteric inhibitor binding sites in GDH. Stimulation of reductive amination through GDH activation reduces DNL and gluconeogenesis. It also augments ketogenesis because of a relative deficiency of TCA cycle intermediates. Stimulation of reductive amination also provides NAD+. The increased NAD+/NADH ratio may activate the Sirt1/AMPK/PGC-1α axis and ultimately correct most metabolic alterations induced by high calorie diets through enhanced fatty acid oxidation and amelioration of insulin resistance. AA, acetoacetate; Ac-CoA, acetyl-CoA; Ala, alanine; α-KG, alpha-ketoglutarate; β-HB, beta-hydroxybutyrate; Cit, citric acid; CS, citrate synthase; FA, fumaric acid; ; FFA, free fatty acid; GDH, glutamate dehydrogenase; Glu, glucose; Glu-6-P, glucose-6-phosphate; Glut, glutamic acid; I-Cit, iso-citric acid; KB, ketone bodies; LA, lactic acid; LD, lactate dehydrogenase; MA, malic acid; Mal-CoA, Malonyl-CoA; MT, mitochondria; OAA, oxaloacetic acid; PA, pyruvic acid; PA-CoA: palmitoyl-CoA; PC, pyruvate carboxylase; PDH, pyruvate dehydrogenase; PEP, phosphoenol pyruvic acid; PEPCK, phosphoenolpyruvate carboxykinase; SA, succinic acid; SUC-CoA, succinyl-CoA; TG, triacylglycerol.

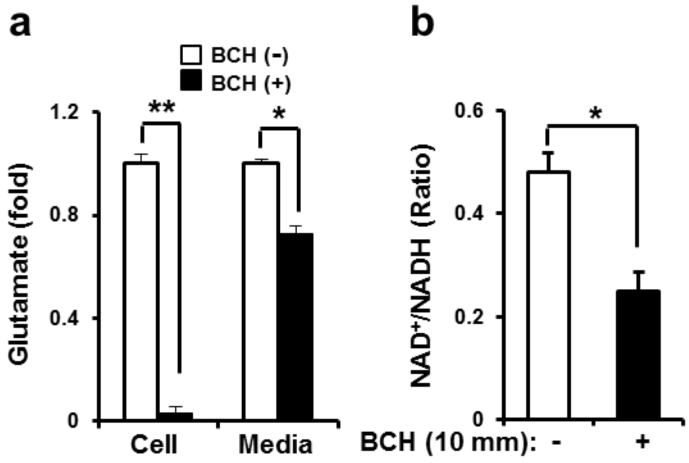

Supplementary Fig. 2. BCH stimulated oxidative deamination in INS-1 beta cells. (A) Glutamate level in cells and culture media was determined using Glutamate Colorimetric Assay Kit (Biovison, Milpitas, CA, USA). (B) NAD+/NADH ratio in cells was determined using NAD+/NADH Quantification Kit (Biovision). $^{*}p<0.05$, $^{**}p<0.01$ vs. BCH-untreated group.

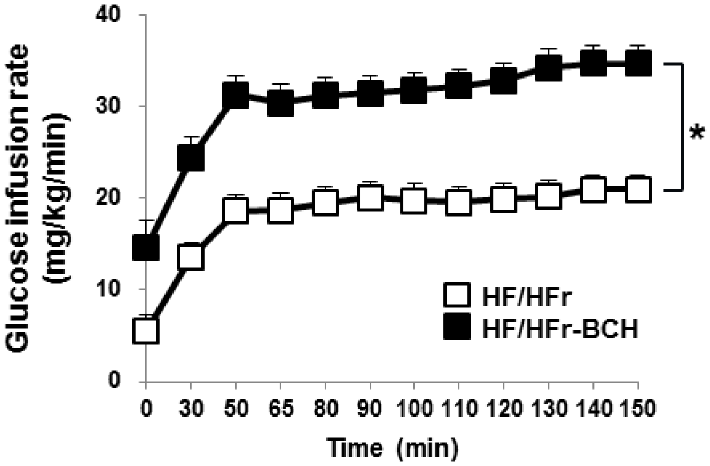

Supplementary Fig. 3. BCH treatment increased glucose infusion rate HF/HFr-fed C57BL/6J mice. Peripheral insulin sensitivity was assessed by hyperinsulinemic–euglycemic clamps. Glucose infusion rates were measured following high fat/high fructose with or without BCH treatment.

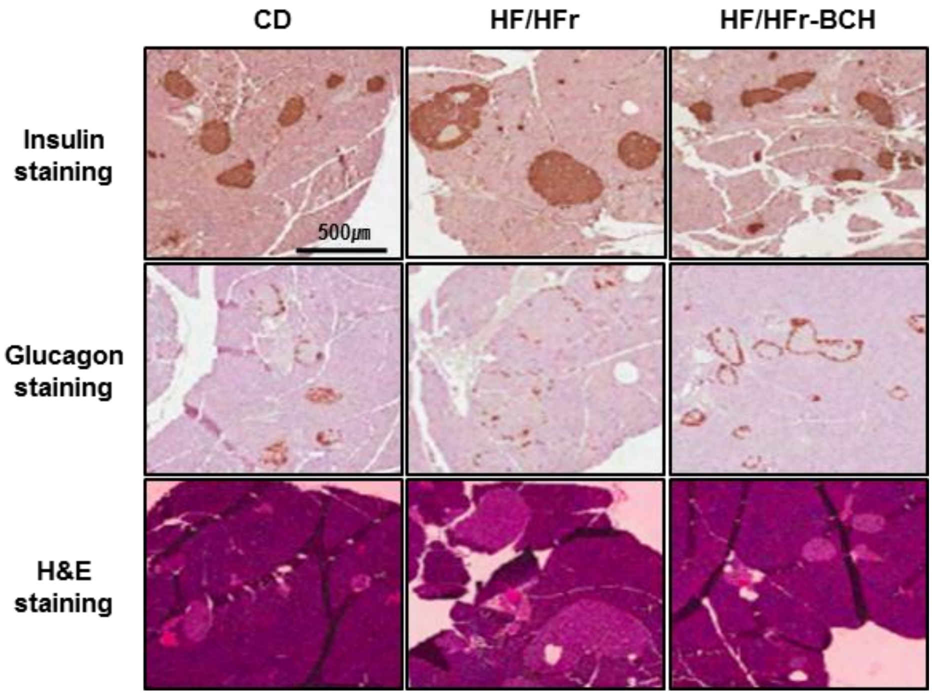

Supplementary Fig. 4. BCH treatment reduced islet hyperplasia in HF/HFr-fed C57BL/6J mice. Effect of BCH on islet hyperplasia was measured by immunohistochemistry. (A) Islets isolated from control diet (CD)-, high fat/high fructose diet (HF/HFr)-, or BCH-injected high fat/high fructose diet (HF/HFr-BCH)-fed mice were stained with anti-insulin antibodies, anti-glucagon antibodies, or hematoxylin and eosin (H&E).

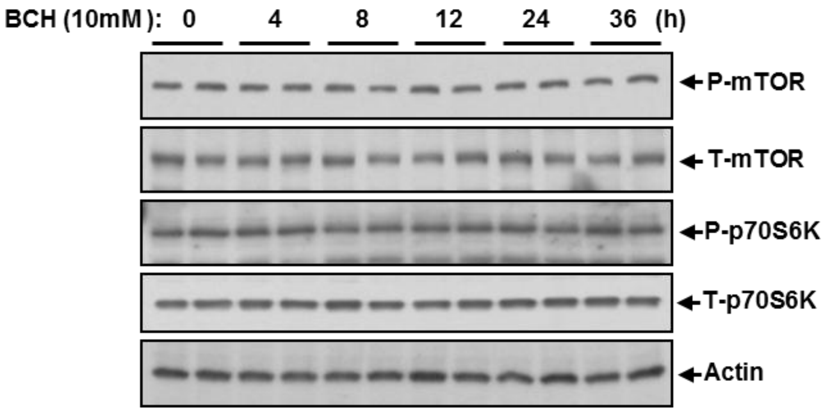

Supplementary Fig. 5. BCH did not reduce P-mTOR level. The levels of amino acid signals (phospho-mTOR and phospho-p70S6 kinase) were determined by immunoblotting with anti-P-mTOR and P-p70S6K antibodies at indicated times after BCH treatment.

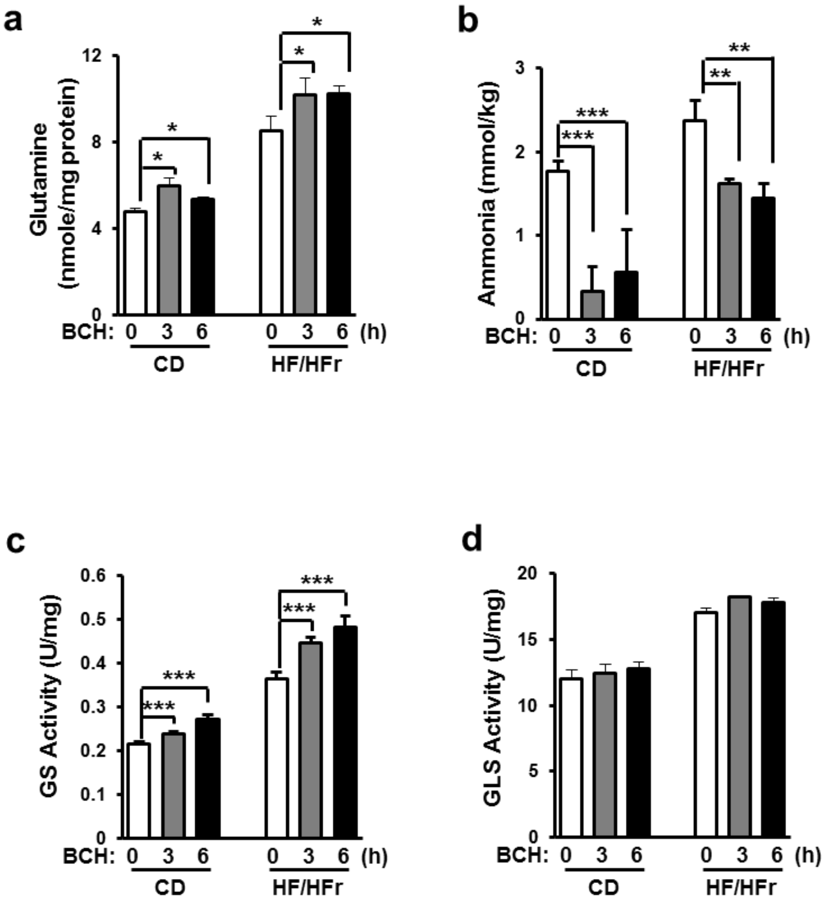

Supplementary Fig. 6. BCH increased the glutamine level and glutamine synthetase activity but decreased the ammonia level in mouse liver extract. We fed a chow diet (CD) or high fat/high fructose (HF/HFr) diet again to CD- or HF/HFr-fed C57BL/6J mice after a 12-h fast. BCH (0.7 g/kg) was administered for 3 or 6 h during re-feeding. The glutamine level from liver extract was measured using a glutamine colorimetric assay kit (Biovision, Milpitas, CA, USA). The ammonia concentration was determined using an ammonia determination kit (Sigma-Aldrich, St. Louis, MO). The activities of glutamine synthetase (GS) and glutaminase were measured using GS and glutaminase microplate activity assay kit (Cohesion Bioscience, London, UK). $^{*}p<0.05$, $^{**}p<0.01$, $^{***}p<0.001$ vs. BCH-untreated group.

Full-length blots for Fig. 2 cropped blots

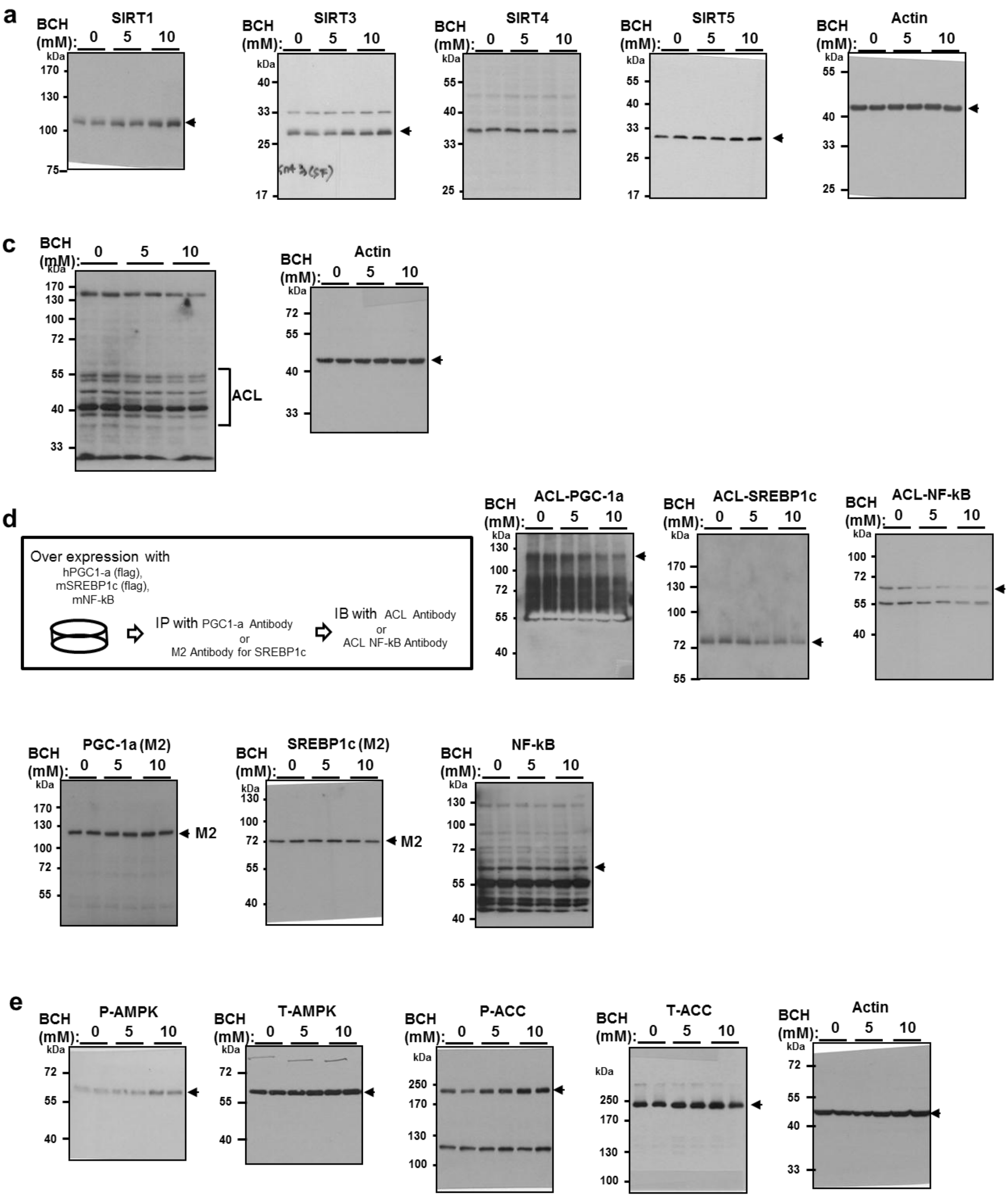

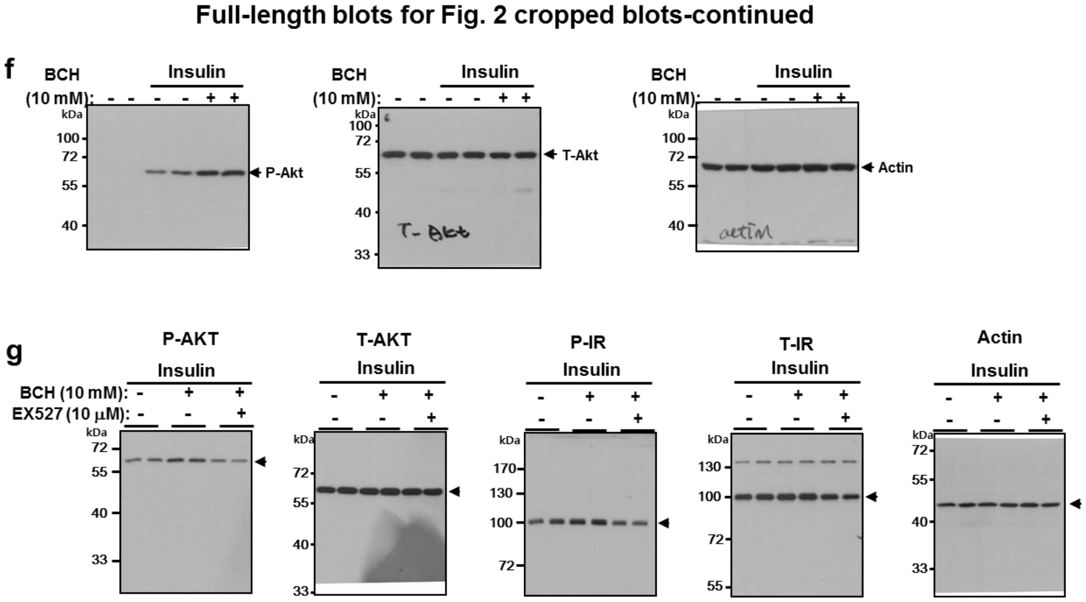

Supplementary Fig. 7. Full-length blots for cropped blots shown in Fig. 2.

Full-length blots for Fig. 5 cropped blots

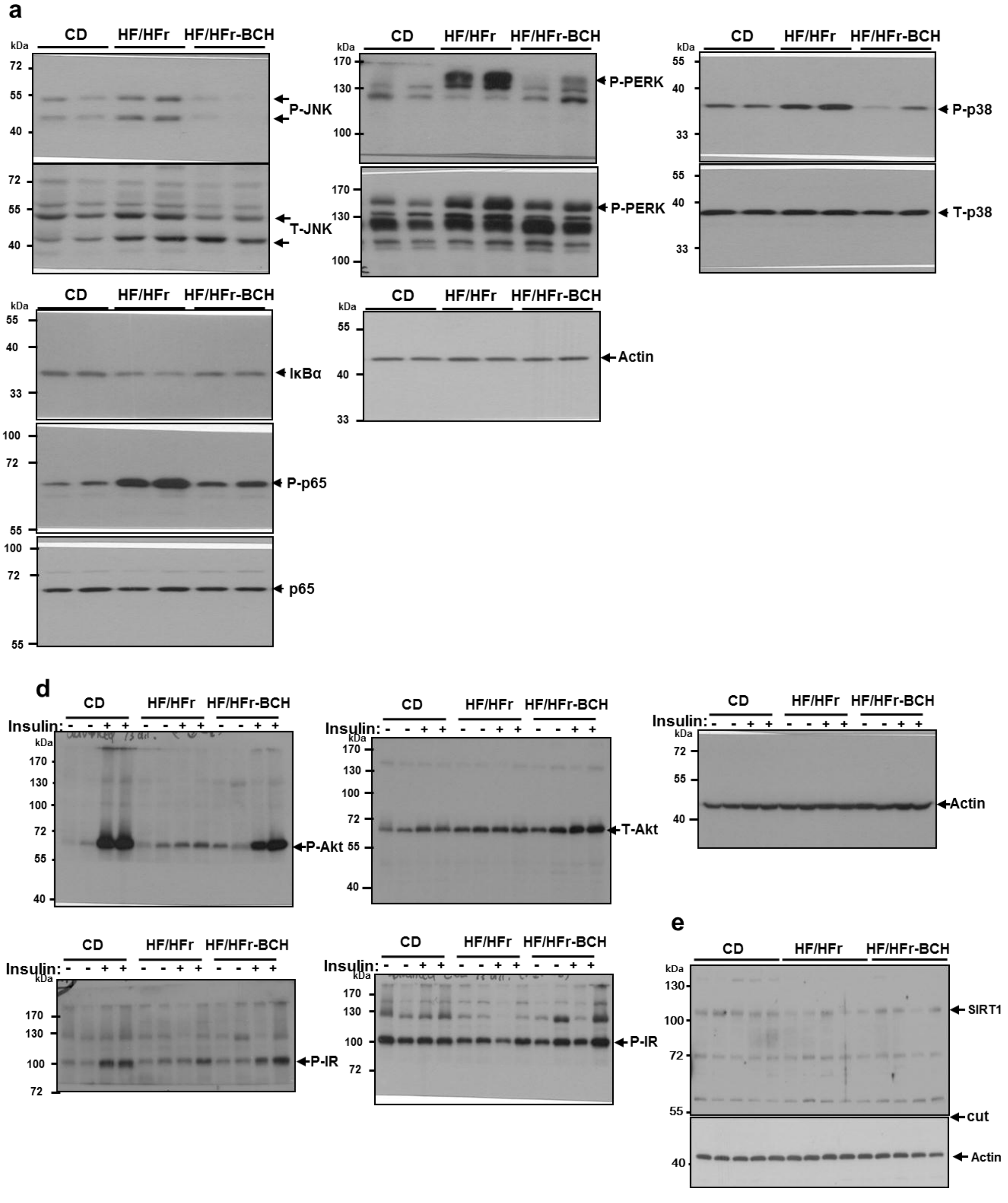

Supplementary Fig. 8. Full-length blots for cropped blots shown in Fig. 5.
